# Isolation and Characterization of Clinical Triazole Resistance *Aspergillus fumigatus* in Iran

**Published:** 2018-07

**Authors:** Faezezeh MOHAMMADI, Seyed Jamal HASHEMI, Seyed Mojtaba SEYEDMOUSAVI, Dorna AKBARZADE

**Affiliations:** 1. Dep. of Medical Parasitology and Mycology, School of Medicine, Qazvin University of Medical Sciences, Qazvin, Iran; 2. Dept. of Medical Mycology and Parasitology, School of Public Hygiene, Tehran University of Medical Sciences, Tehran, Iran; 3. Food Microbiology Research Center, Tehran University of Medical Sciences, Tehran, Iran; 4. Dept. of Medical Microbiology, Radboud University Medical Center, Nijmegen, Netherlands; 5. School of Pharmacy, Tehran University of Medical Sciences, Tehran, Iran

**Keywords:** Azole resistance, *Aspergillus fumigatus*, Iran

## Abstract

**Background:**

*Aspergillus fumigatus* is a major cause of allergic syndromes, aspergilloma and life-threatening invasive infections in immunocompromised hosts. To date, a wide range of mutations in *A. fumigatus*have been described conferring azole-resistance, which commonly involves modifications in the *cyp51A*-gene (substitutions at codons G54, G138, P216, F219, M220, G448 and specifically codon L98 in combination with a 34-bp tandem repeat in the promoter region of the gene), the target for azole antifungals. We investigated the prevalence of azole-resistance in clinical *A. fumigatus* isolates obtained from patients in Iran during 2010 to 2014.

**Methods:**

Overall, 172 clinical *A. fumigatus* isolates obtained from patients with underlying disease including transplantation, granulocytopenia, chronic liver disease, chronic obstructive pulmonary disease (COPD) and allergic bronchopulmonary aspergillosis (ABPA). Samples were collected between Jan 2009 and Nov 2014 from five provinces of Iran (Tehran, Shiraz, Isfahan, Khorasan razavi and East Azerbaijan). Antifungal susceptibility test was determined according to EUCAST reference method for itraconazole (ITC), voriconazole (VRC) and posaconazole (POS). All isolates were confirmed by amplification of the partial tubulin gene.

**Results:**

Of 172 *A. fumigatus* isolates tested, six isolates (3.5%) had high MIC values of ITC (>16 mg/L) and VRC (≥4 mg/L). All six isolates showed a multi-resistant phenotype with high MICs of ITC and VRC.

**Conclusion:**

We determined in-vitro susceptibility a profile of 172 clinically isolates of *A. fumigatus* against triazole in Iran. Azole-resistance is an emerging problem in *A. fumigatus* and international surveillance is warranted.

## Introduction

Invasive aspergillosis (IA) has increased in recent decades. *Aspergillus fumigatus* is one of the most common invasive aspergillosis associated with significant morbidity and mortality in the immunocompromised host such as solid organ, hematopoietic stem cell transplant recipients and patients receiving chemotherapy (1, 2).

Present treatment options of IA include three classes of antifungal agents: polyenes (amphotericin B), echinocandins (caspofungin) and triazoles (Itraconazole (ITC), Voriconazole (VRC), Posaconazole (POS)) (3, 4). Azole resistance in clinical *A*. *fumigatus* isolates is important and a number of *A. fumigatus* isolates with in vitro ITC and VRC resistance have been reported over the recent years (5–8).

The azole antifungal inhibits the ergosterol biosynthesis pathway via the inhibition demethylation of sterol 14-alpha-demethylase (*CYP51*) (9). Two distinct pathways of azole resistance have been reported: first in patients with aspergilloma and chronic pulmonary aspergillosis with long-term treatment with medical azoles; second in the environment through the exposure of the fungus to the azole fungicides used in agricultural products (10, 11).

The *cyp51A* gene is the target of azole agents and azole resistance is commonly due to mutations in the *cyp51A* gene (12, 13). Different single nucleotide polymorphisms (SNPs) such as codons 54, 98 and 220 in *cyp51A* gene have been described in clinical strains correlated with triazole resistance but the most frequently reported resistance mechanism is an alteration at codon 98 (L98H), in combination with 34 base pair tandem repeat in the promoter region (TR34/L98H) (14). The Antifungal susceptibility tests have been described for the detection of azole resistance in *Aspergillus* spp. by the Clinical and Laboratory Standards Institute (CLSI) and the European Committee on Antibiotic Susceptibility testing (AFST-EUCAST) for molds (15, 16). Unfortunately **in vitro** antifungal susceptibility **testing** for clinical isolates of *A. fumigatus* is not routinely performed at present clinical microbiology laboratories in Iran.

In the present study, we determined in-vitro susceptibility profiles of 172 clinically isolates of *A. fumigatus* against three antifungal drugs, including itraconazole, voriconazole, and posaconazole.

## Materials and Methods

### Collection of samples and identification

One hundred and seventy-two clinical *A. fumigatus* isolates obtained from the lower respiratory tract (n=82; 47.67%), sinus (n=39; 22.67%), abscess (n=23; 13.37%), biopsy samples (n=18; 10.46%) and cerebrospinal fluid (n=10; 5.81%). Samples were collected between Jan 2009 and Nov 2014 by routine diagnostic procedures at hospital laboratories in 5 provinces of Iran (Tehran, Shiraz, Isfahan, Khorasan Razavi and East Azerbaijan). The isolates were identified by macroscopic colony morphology andmicroscopic morphology of conidia growing on the Sabouraud dextrose agar plate (SDA; Difco). Afterwards confirmed by amplification of the partial tubulin gene using the primer set Bt2a (5′GGTAACCAAATCGGTGCTGCTTTC3′) and Bt2b (5′ACCCTCAGTGTAGTGACCCTTGGC3′) (17, 18).

### The study was approved by Ethics Committee of the university.

#### DNA extraction

DNA was extracted as described previously (20). In brief, the isolates were cultured on Sabouraud Dextrose Agar (SDA). A loop full of a fresh colony was harvested and added to 200 μl of lysis buffer (100 mM NaCl, 10 mM Tris-HCl, pH 8, 2% Triton X-100, 1% sodium dodecyl sulfate, 1mM EDTA, pH 8) with 0.1-g glass beads (diameters, 0.4 to 0.6 mm). After shaking by vortexing, the conidia were incubated at 70°C for 30 min while they were shaken. Then, 200 μl of phenol-chloroform-isoamyl alcohol (25:24:1) saturated with pH 8.0 aqueous buffer was added, and the samples were incubated for 5 min while they were shaken. After centrifugation for 5 min, the upper phase containing the DNA was transferred to a new tube and was kept at −20 °C until use.

#### PCR amplification

PCR amplification was carried out in a final volume of 50 μl. Each reaction contained 0.5 μl of template DNA, 1 μl of each primer (Bt2a and Bt2b),1 μl of dNTPs 5x, 10 μl of 5 x HF Buffer, and 1.25U of *Taq* polymerase (Roche Molecular Biochemicals, Mannheim, Germany). An initial denaturation step at 98°C for 30 sec was followed by 35 cycles of denaturation at 98°C for 10 sec, annealing at 61°C for 30 sec, and extension at 72°C for 1 min, with a final extension step of 72°C for 10 min (19).

#### In vitro Susceptibility testing

All 172 isolates of *A. fumigatus* were subculture from the primary culture onto a four-well agar plate containing 4 mg/L of ITC, 1 mg/L of VRC and 0.5 mg/L of POS. The ability to grow was evaluated after 48 h. Any isolate that grew on one of the azole-containing media was stored and selected for Susceptibility patterns of ITC (Jenssen Pharmaceutical, Beerse Belgium), VRC (Pfizer, United Kingdom) and POS (Merck & Co., Inc., NJ, USA) using a broth microdilution test, according to the European Committee on Antibiotic Susceptibility Testing (EUCAST) reference method (20). The MIC (minimum inhibitory concentration) endpoints were recorded after 48h and finally, the MIC was defined as the lowest antifungal concentration produced a complete inhibition with a reading mirror. A putative resistance breakpoint of >2 mg/L was used for ITC, VRC and >0.5 mg/L for POS. Stock solutions of the drugs were dissolved in dimethyl sulfoxide in each well and stored at −70°C until being used. Itraconazole, voriconazole, andposaconazole were serially diluted to provide a final drug concentration range of 16 to 0.008 μg/m. Prepare inoculum suspensions from fresh cultures with sterile saline and 0.05% tween 80.Afterward homogenize the suspension for 15 sec with vortex mixer at 2000 rpm. The turbidity of the conidial spore suspensions was measured at 530 nm to obtain a final inoculum of 2–5×10^6^ cfu/mL and dilute the conidial suspension 1:10 with sterile distilled water for final working inoculum of 2–5 ×10^5^cfu/mL inoculated each well. Spore suspensions were loaded into flat-bottomed microtitre plates and incubated at 37 °C for 48 h. Susceptibility tests were performed at least three times with each strain on different days. *Paecilomyces variotii* (ATCC 22319), *Candida parapsilosis* (ATCC 22019), and *C. krusei* (ATCC 6258) were used for quality controls in all experiments (21).

## Results

Overall, 172 clinical *A. fumigatus* isolates obtained from patients with underlying disease including transplantation (n=58; 33.72%), granulocytopenia (n=42; 24.41%), chronic liver disease (n=31; 18.02%), chronic obstructive pulmonary disease (COPD) (n=23; 13.37%) and allergic broncho pulmonary aspergillosis (ABPA) (n=18; 10.46%). Samples were collected from five provinces of Iran including Tehran (n=93; 54.06%), Shiraz (n=34; 19.76%), Khorasan Razavi (n=25; 14.53%), Isfahan (n=15; 8.72%), and East Azerbaijan (n=5; 2.9%).

### Antifungal susceptibility tests

**Fig. 1:**
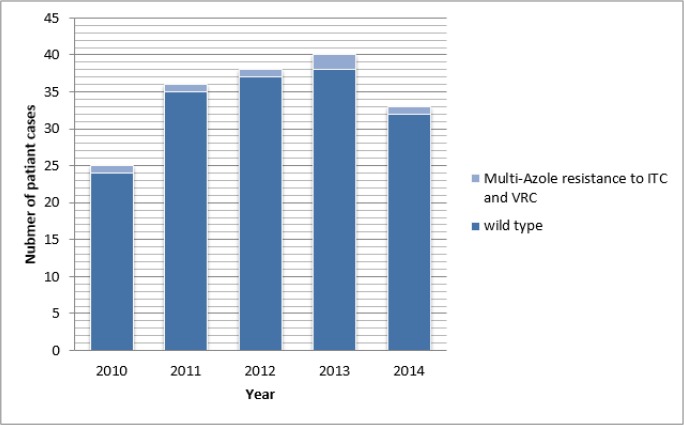
Azole resistance frequency of *A. fumigatus* isolates from patient 2010–2014

Of the 172 *A. fumigatus* isolates, 6 isolates (recovered from separate patients) grew on the wells containing ITC and VRC indicating a prevalence of 3.5% (Table 1). All six isolates presenting azole-resistance phenotype were identified as *A. fumigatus* by sequence analysis of partial β-tubulin gene. The origin and history of previous azole exposure of 6 clinical *A. fumigatus* isolate with high MICs of ITC and VRC are displayed in (Table 2). All sixisolates showed a multi-resistant phenotype with high MICs of ITC (>16 mg/l) and VRC (≥2 mg/l) with MIC range of 0.0063->16 for ITC and MIC range of 0.031–8 for VRC (Fig. 1). Five of these isolates were obtained from patients with chronic pulmonary aspergillosis (CPA) and one from a patient with allergic bronchopulmonary aspergillosis (ABPA).

**Table 1:** Origin and in vitro susceptibilities of 6 clinical *Aspergillus fumigates* isolates

***Azole-resistant isolates***	***MIC(mg/L)***		
	***ITC***	***VRC***	***POS***
*Aspergillus fumigatus* T-IR-AF 1002	≥16	4.0	0.25
*Aspergillus fumigatus* T-IR-AF 1088	≥16	2.0	0.5
*Aspergillus fumigatus* T-IR-AF 1143	≥16	8.0	0.5
*Aspergillus fumigatus* T-IR-AF 1416	≥16	8.0	0.5
*Aspergillus fumigatus* T-IR-AF 1499	≥16	4.0	0.5
*Aspergillus fumigatus* T-IR-AF 1521	≥16	8.0	0.5

**Table 2:** The history of previous azole exposure and underlying azole resistance of six clinical *Aspergillus fumigatus* isolates (Chronic Pulmonary Aspergillosis (CPA), Allergic Bronchopulmonary Aspergillosis (ABPA))

***Azole-resistant isolates***	***Disease***	***Previous Azole exposure***
*Aspergillus fumigatus* T-IR-AF 1002	CPA	Yes
*Aspergillus fumigatus* T-IR-AF 1088	CPA	Yes
*Aspergillus fumigatus* T-IR-AF 1143	CPA	Yes
*Aspergillus fumigatus* T-IR-AF 1416	CPA	Yes
*Aspergillus fumigatus* T-IR-AF 1499	ABPA	Yes
*Aspergillus fumigatus* T-IR-AF 1521	CPA	Yes

## Discussion

We showed 3.5% prevalence azole-resistant *A. fumigatus* isolates obtained from patients underlying disease in Iran over the recent 5 years (2010 to 2014). There was notsignificant increase in the prevalence of azole-resistant *A. fumigatus* compared with previous study in Iran from 2003 to 2009 (22).

The prevalence of azole-resistant *A. fumigatus* was determined in Iran remarkably increased from 3.3% to 6.6%. In this study, TR34/L98H mutations in the *cyp51A* gene were the most prevalent among the resistant isolates (6). Resistance to the triazole antifungal agents is an emerging public health problem among clinical isolates of *Aspergillus* spp. (23).

The main mechanism of azole resistance in *A. fumigatus* was associated with the modification of the 14-sterol demethylase target enzyme (*cyp51*) by several mutations in the cyp51A-gene (14, 24). In vitro survey of Azole resistance in *A. fumigatus*is linked to mutations in both the *cyp51A* and the *cyp51B* genes and to the over expression of several drug efflux transporters (23, 25, 26).

Multi azole resistance in *A. fumigates* is typically associated with point mutations in cyp51A gene, especially TR34/L98H mutation has become an emerging problem and is increasingly recognized as a cause of treatment failure (10, 27, 28).

Our findings showed multi azole resistance to occur in patients previously exposed to azoles. Long-term of drug exposure and use of azole compounds in the environment are important factors in the selection of resistance and increases in cross-resistance of *A. fumigatus* isolates to the triazoles would be damaging to the clinical treatment (29, 30). Snelders et al. indicated relationship between environmental azole use and development of cross-resistance to medical triazoles (31). This may suggest an alternative route of resistance development through exposure to triazole fungicide compounds in the environment. The activities of ITC and VRC were compared against 338 Spanish clinical isolates of *Aspergillus* spp. and found among the 12 isolates for which the itraconazole MIC was 4 g/ml, the voriconazole MIC was 2 g/ml for 8 isolates), suggesting some degree of cross-resistance (32). Cross-resistance to ITC and POS had been associated with G54 mutants, while G448 mutants display cross-resistance to voriconazole and ravuconazole (33–36). The role of the M220 substitutions was detected in multiazole resistance phenotype to all triazole agents in five clinical isolates of *A. fumigatus* (35). Recent studies of multiazole resistance clinical isolates in the Netherlands showed that TR34/L98H mutation is the most comm. Only conferring azole resistance in the cyp51A gene (11, 31, 37). The *A. fumigatus* isolates harboring TR34/L98H mutation were cultured from soil and compost and shown genetic relatedness to clinical resistant isolates (11).

## Conclusion

All six resistance isolates were obtained from patients with a history of exposure to azoles that suggest the development of azole-resistance due to exposure of an individual patient to azole therapy. Of note, five out of six azole-resistant isolates were recovered from CPA patients that require long-term maintenance antifungal therapy to improve symptoms. Long-term azole therapy may increase the risk of resistance and may result in different levels of resistance to the various azoles.

## Ethical considerations

Ethical issue principles including plagiarism, informed consent, misconduct, data fabrication and/or falsification, double publication and/or submission, redundancy, etc. have been completely observed by the authors.
